# Enhancing Esthetics With Digital Dentistry: A 2‐Year Follow‐Up of 3D‐Printed Restorations

**DOI:** 10.1111/jerd.13491

**Published:** 2025-06-01

**Authors:** Cristian Higashi, Rafaelly Cubas Camargo, Stephanie Gomes Assunção Alves, Carlos de Oliveira Viana Correia, Pedro Hideki Hiromoto

**Affiliations:** ^1^ Latin American Dental Research and Teaching Institute Curitiba Brazil; ^2^ Medicine and Life Science School Pontifical Catholic University of Paraná Curitiba Brazil

**Keywords:** dental esthetics, dental materials, dental technology, dental veneers, three‐dimensional printing

## Abstract

Esthetics are a common motivation for dental treatments, with veneers, both direct and indirect, being popular restorative options. Technological advancements, particularly in additive manufacturing (3D printing), have streamlined results. In dentistry, 3D printing is used to fabricate restorations ranging from simple inlays to complex crowns and maxillofacial prostheses.

**Objective:**

The objective of this study is to present a case in which indirect resin veneers were fabricated using additive manufacturing technology to enhance the smile of a 24‐year‐old female patient.

**Clinical Considerations:**

The patient sought treatment for esthetic concerns related to small and yellowish maxillary anterior teeth. The treatment plan involved in‐office bleaching followed by digital smile design, intraoral mock‐up, and the fabrication of indirect veneers using additive manufacturing.

**Conclusions:**

It was concluded that the combination of digital planning and intraoral mock‐up provides predictability in treatment planning. Additionally, additive manufacturing techniques can be employed in various stages of the restorative procedure, making it more efficient and promoting satisfactory esthetic outcomes with clinical stability observed after two years.

## 1 Introduction

Esthetics plays an increasingly significant role in dentistry, with the desire for a more harmonious smile being one of the primary motivations for patients seeking treatment [1, 2]. In this context, dental interventions that replicate the natural characteristics of teeth have become a central focus of clinical practice [3]. To meet this demand, new tools and restorative techniques are continually developed, expanding the range of available therapeutic options. For anterior teeth, where esthetics are crucial, conservative treatments performed either directly or indirectly are often preferred [4]. In this scenario, dental veneers have been widely used. Their indications include teeth discolored by tetracycline, fluorosis, amelogenesis imperfecta, or aging, as well as the restoration of fractured, worn, abnormally shaped, or slightly misaligned teeth. Veneers can be fabricated using different methods [5, 6].

The advancement of digitalization, combined with the introduction of computer‐aided design and manufacturing (CAD/CAM) technologies in dentistry, has provided new clinical and laboratory protocols for the fabrication of restorations [7]. Until recently, the process of fabricating indirect restorations predominantly relied on subtractive manufacturing, which produces restorations from raw blocks or discs by milling with specific tools. This method reduces production time and technical work compared with other fabrication techniques [8, 9]. However, in recent decades, additive manufacturing, also known as 3D printing, has been introduced as a beneficial alternative, allowing for the production of complex structures with greater efficiency [10].

In dentistry, 3D printing is employed to fabricate restorations ranging from simple inlays to complex crowns and maxillofacial prostheses [11]. The restoration fabrication process is based on the successive deposition of layers of photosensitive material (liquid resins), which are partially polymerized in a vat and later subjected to polymerization in a specific curing unit. During the process, the printer's build platform moves vertically to enable the formation of each layer. The upward movement of the platform stirs the liquid resin, which must level out before the platform descends again into the resin vat [12, 13]. This technique offers significant advantages such as high precision, fast production, reduced material waste, and lower costs compared with subtractive methods [14, 15] However, its application in dentistry is highly dependent on the available materials, which must meet requirements for biocompatibility, mechanical strength, and dimensional accuracy. Additionally, the resins used must exhibit appropriate rheological and optical characteristics for the technique, ensuring efficiency and quality in the results [13].

Thus, this article presents a clinical case report describing the fabrication of resin veneers using additive manufacturing to achieve aesthetic rehabilitation of multiple anterior teeth.

## 2 Case Report

**FIGURE 1:**
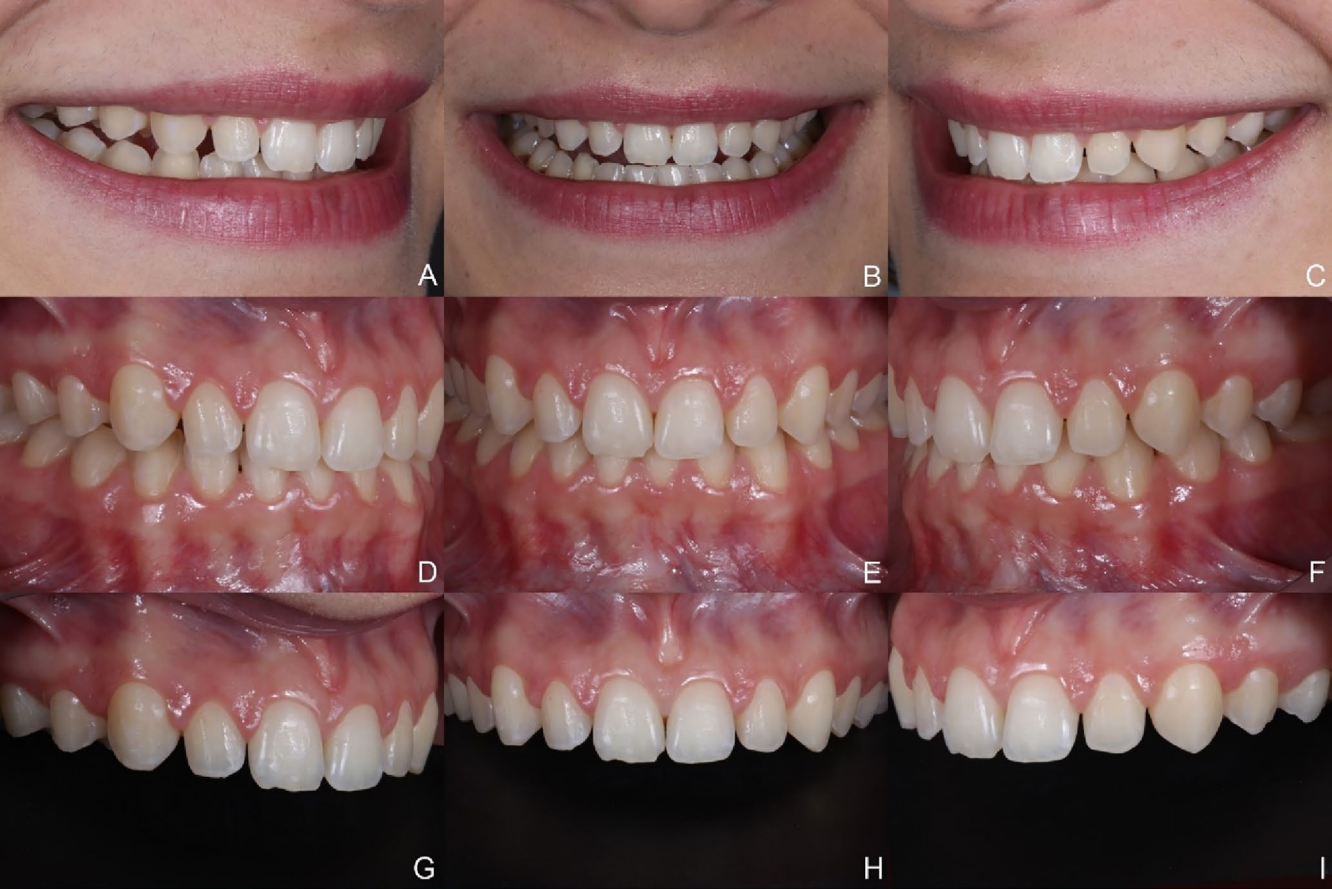
(A to I). Initial extraoral and intraoral photographs. Small and yellowish maxillary anterior teeth are observed.

A 24‐year‐old female patient sought dental treatment, reporting dissatisfaction with the esthetics of her smile due to small and yellowish maxillary anterior teeth. Clinical examination revealed a disharmonic smile with unfavorable dental proportions, minor incisal irregularities, and small diastemas between the maxillary central and lateral incisors, as well as between the lateral incisors and canines (Figure 1). After obtaining written informed consent for the use of images in this case report, and following a detailed case analysis, the proposed treatment plan consisted of dental bleaching followed by re‐anatomization of the teeth using indirect veneers fabricated through additive manufacturing, involving second maxillary premolar to second maxillary premolar.

### 2.1 In‐Office Bleaching

Initially, in‐office bleaching was performed using 35% hydrogen peroxide (Whiteness HP, FGM, Joinville, Brazil). The initial tooth shade was recorded using a shade guide (VITA Classical, Vita Zahnfabrik, Bad Säckingen, Germany). A lip retractor (Lip Expand, Indusbello, Londrina, Brazil) was positioned, and the gingival tissue was protected with a gingival barrier (Top Dam Blue, FGM, Joinville, Brazil). The bleaching gel (Whiteness HP, FGM, Joinville, Brazil) was applied in three 15‐min applications, totaling 45 min per session. Following each application, the gel was removed by aspiration and the teeth were thoroughly rinsed with water. The gingival barrier and lip retractor were subsequently removed. Three bleaching sessions were performed at weekly intervals. Post‐treatment evaluation of tooth color was conducted 2 weeks after the completion of the bleaching protocol.

### 2.2 Smile Design and Intraoral Mock‐Up

**FIGURE 2:**
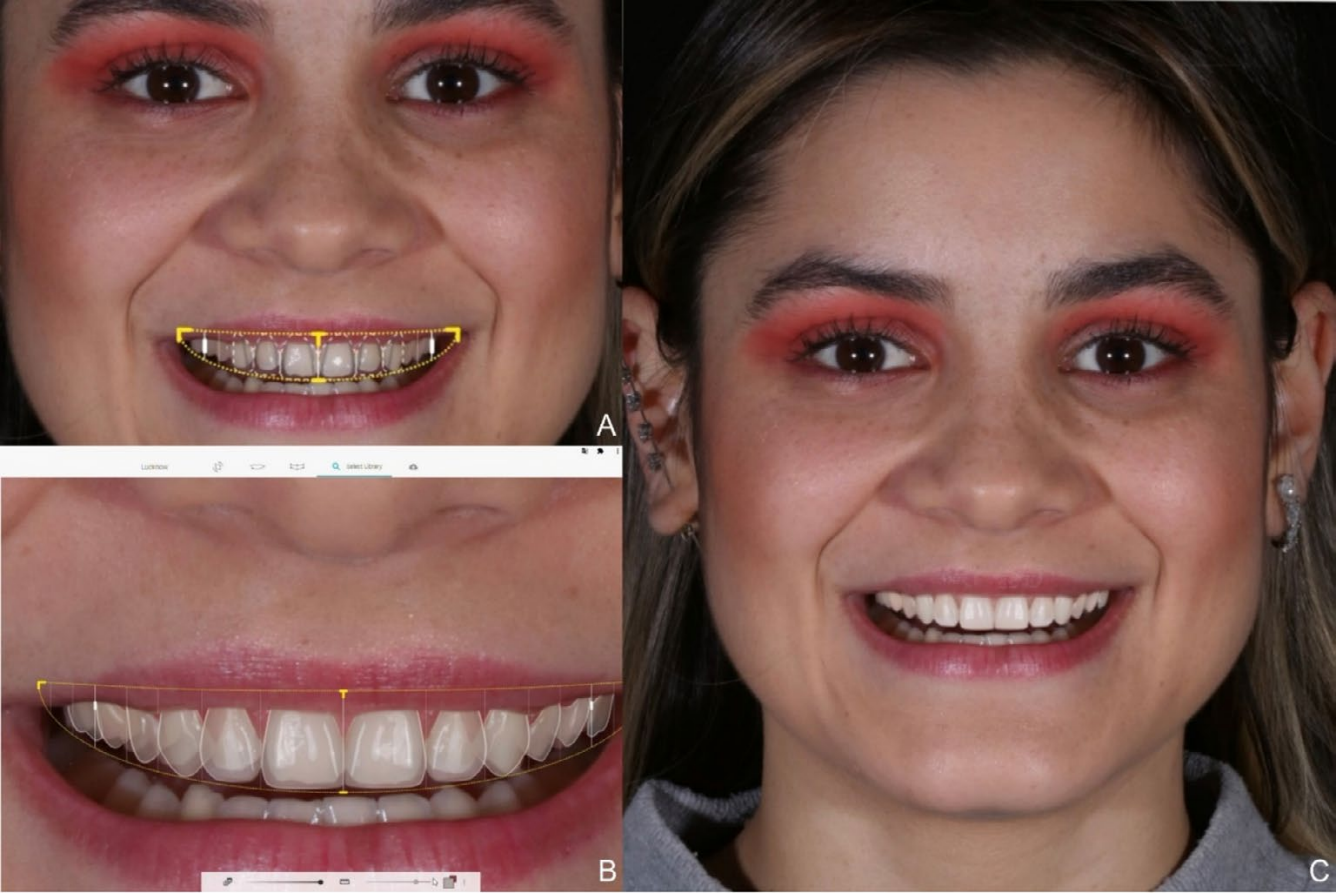
Smile design planning performed using the SmileCloud software, providing a precise digital visualization of the proposed changes.

**FIGURE 3:**
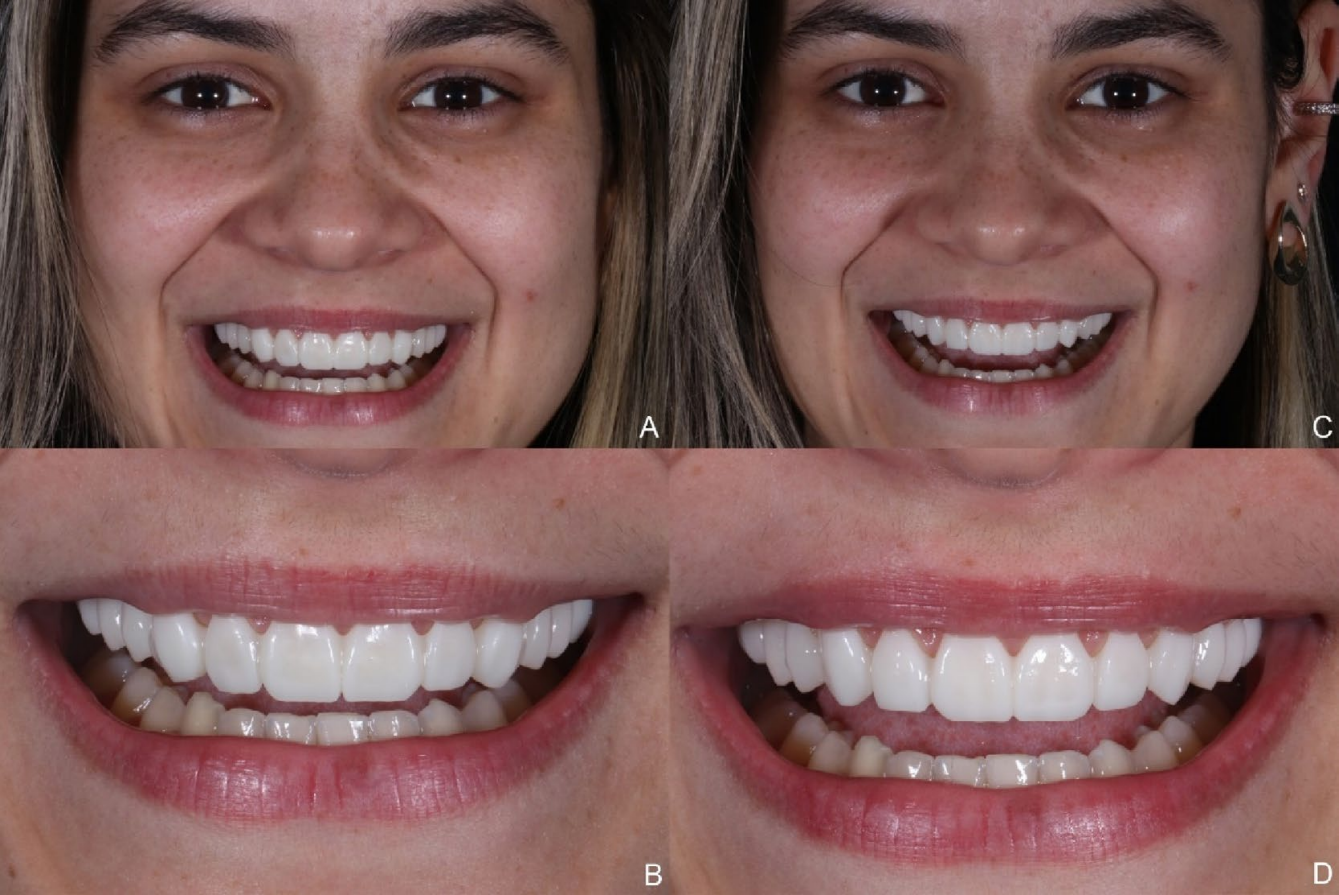
Mock‐ups of the two planned designs, printed using the 3D mockup shell technique. (A, B). Design 1. (C, D). Design 2.

Two weeks post‐bleaching, the second phase of treatment commenced. For restorative planning, digital facial, extraoral, and intraoral photographs were taken, and the arches were scanned using an intraoral scanner (3Shape Trios 3 Pod, 3Shape, Copenhagen, Denmark). Digital smile planning was performed using specialized software (SmileCloud, Dentcof, Timișoara, Romania) and dental libraries available within the program, based on facial reference lines (Figure 2). Two esthetic mock‐up options with distinct designs were fabricated using additive manufacturing with a resin designed for 3D printing of temporary crowns in shade B1 (priZma 3D Bio Prov, Makertech Labs, Tatuí, Brazil). These prototypes were tested on the patient's teeth, allowing the selection of her preferred design and enabling functional and esthetic evaluation by the clinician (Figure 3).

### 2.3 Dental Preparations and Digital Impression

**FIGURE 4:**
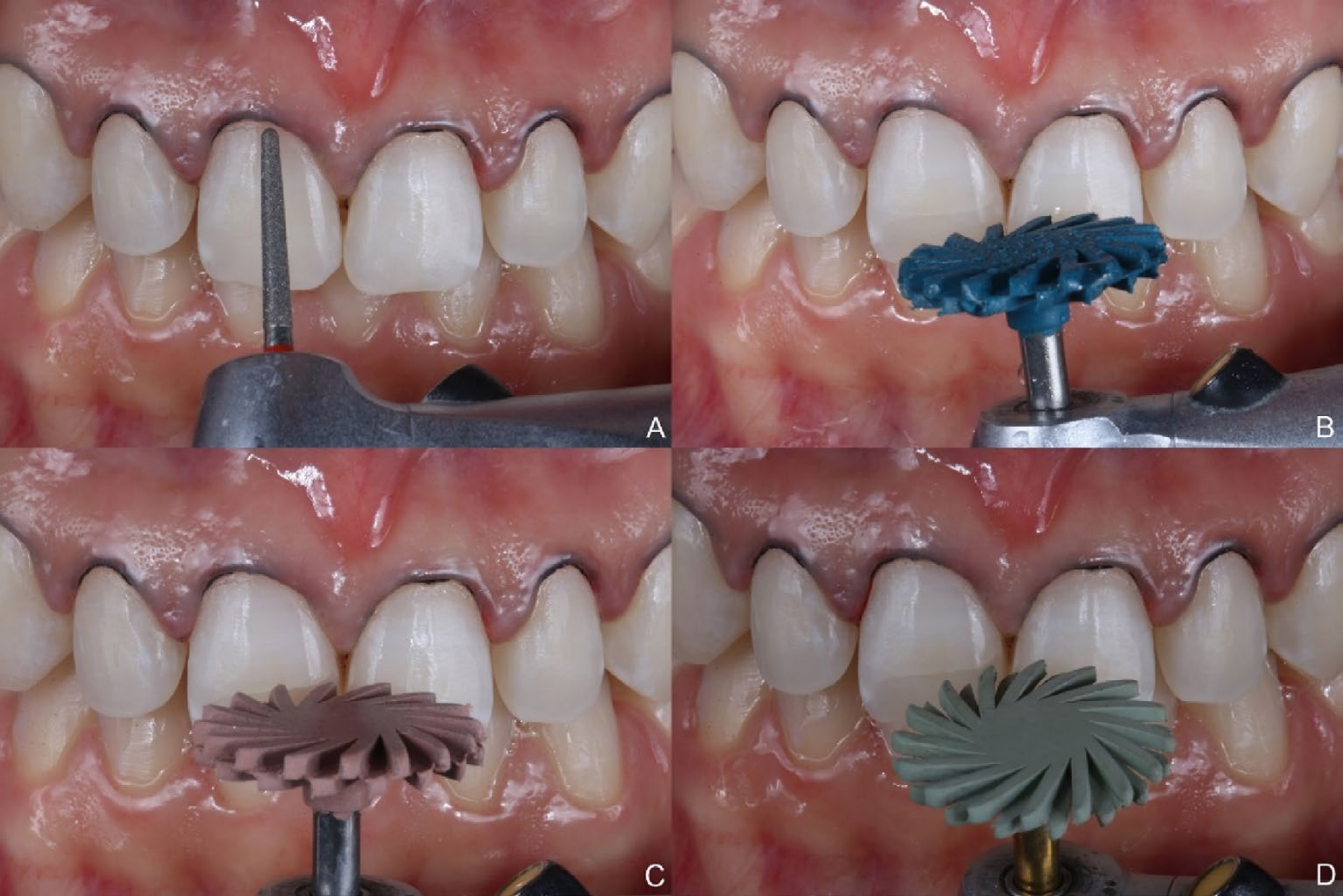
Dental preparations following a minimally invasive protocol, prioritizing the preservation of natural dental structures. (A) Fine‐grit, rounded diamond bur used on the buccal and incisal surfaces to regularize edges and create an adequate emergence profile. (B–D) Preparations polishing performed using a sequence of diamond‐impregnated rubber wheels.

**FIGURE 5:**
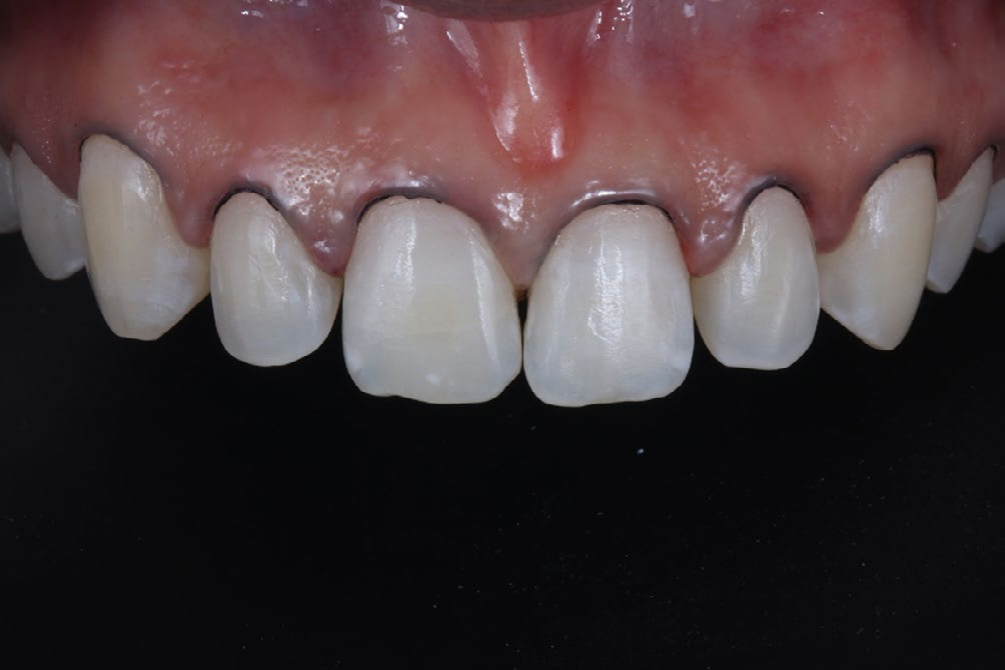
Final aspect of the dental preparation, ready for intraoral scanning.

Teeth #4 to #14 underwent minimally invasive preparations. Retraction cords (Ultrapak #000, Ultradent, Indaiatuba, Brazil) were placed in the gingival sulcus, and a fine‐grit, rounded diamond bur (#2135F, KG Sorensen, São Paulo, Brazil) was used on the buccal and incisal surfaces to regularize edges and create an adequate emergence profile. Preparations were polished using a sequence of diamond‐impregnated rubber wheels (Twist Gloss Spiral, American Burrs, Palhoça, Brazil) (Figures 4 and 5). Digital scanning of the prepared teeth was performed (3Shape Trios 3 Pod, 3Shape, Copenhagen, Denmark), and color selection was conducted using the VITA Classical (VITA Classical, Vita Zahnfabrik, Bad Säckingen, Germany) shade guide, accompanied by photographic documentation.

### 2.4 Laboratorial Procedures

**FIGURE 6:**
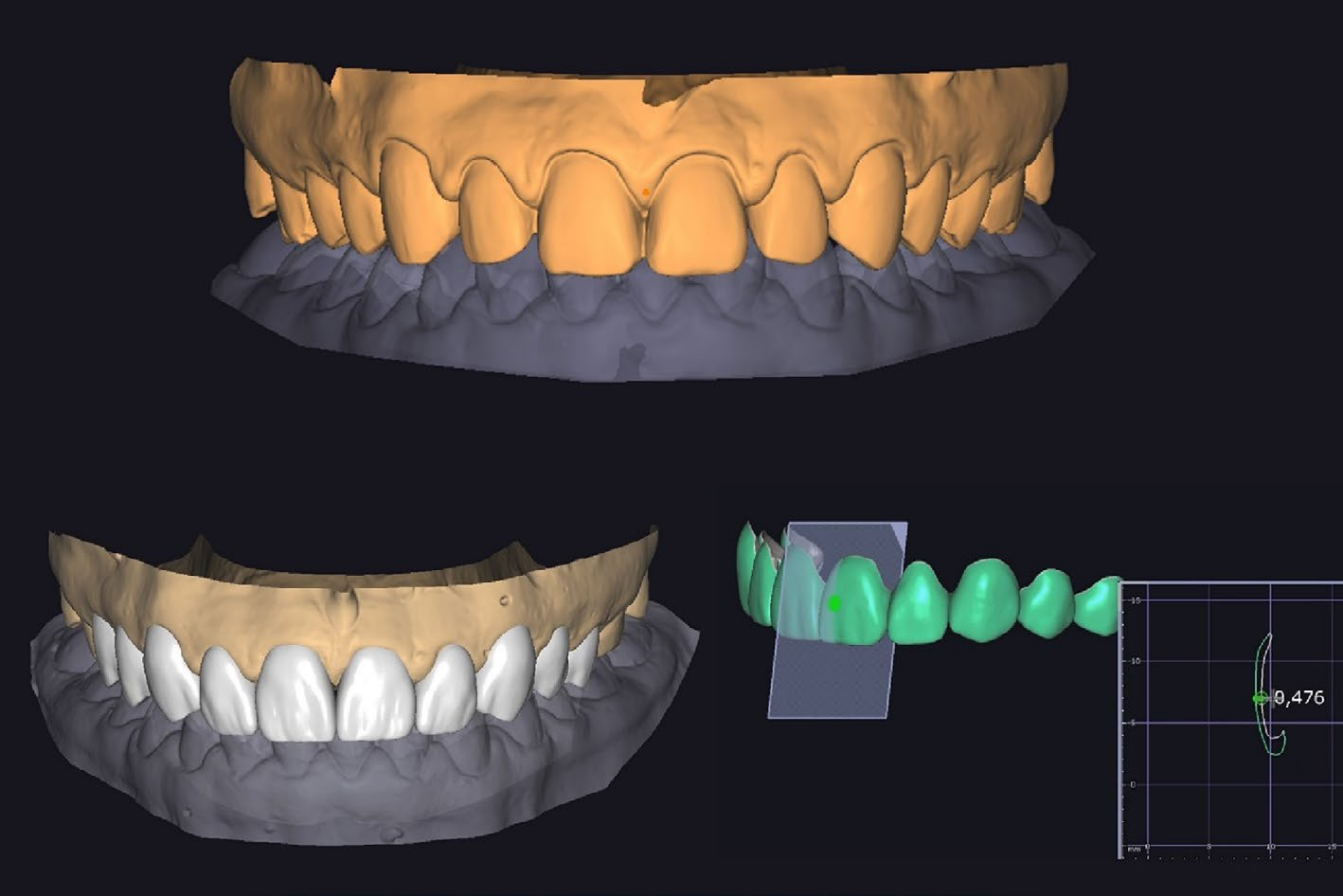
Definitive restoration planning performed using the DentalCAD design software, ensuring precision in design and functional and esthetic integration.

**FIGURE 7:**
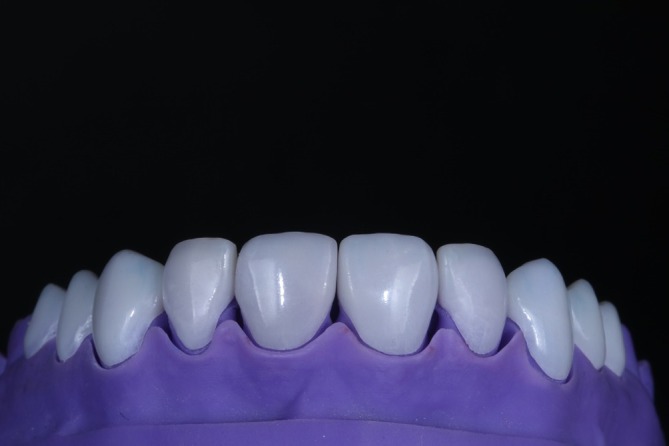
Definitive resin restorations fabricated using priZma 3D Bio Crown resin with 3D printing, offering a personalized and aesthetic solution.

The indirect restorations were fabricated according to the initially selected design using additive manufacturing. The veneers were designed with specific software (DentalCAD, Exocad, Darmstadt, Germany), achieving a thickness of ~0.4–0.7 mm (Figure 6). Printing was carried out using a biocompatible nanohybrid composite resin with silanized ceramic and zirconia components, appropriate for 3D printing (priZma 3D Bio Crown, Makertech Labs, Tatuí, Brazil). The restorations underwent post‐processing, which included cleaning with isopropanol alcohol for 5 min, followed by post‐polymerization in a UV‐polymerization machine for 30 min. Finally, they were adjusted and finished with a UV‐curable glaze (priZma SEAL, Makertech Labs, Tatuí, Brazil) to reduce porosities, seal the resin surface, enhance abrasion resistance, and provide the desired gloss (Figure 7).

### 2.5 Try‐In and Bonding Procedures

**FIGURE 8:**
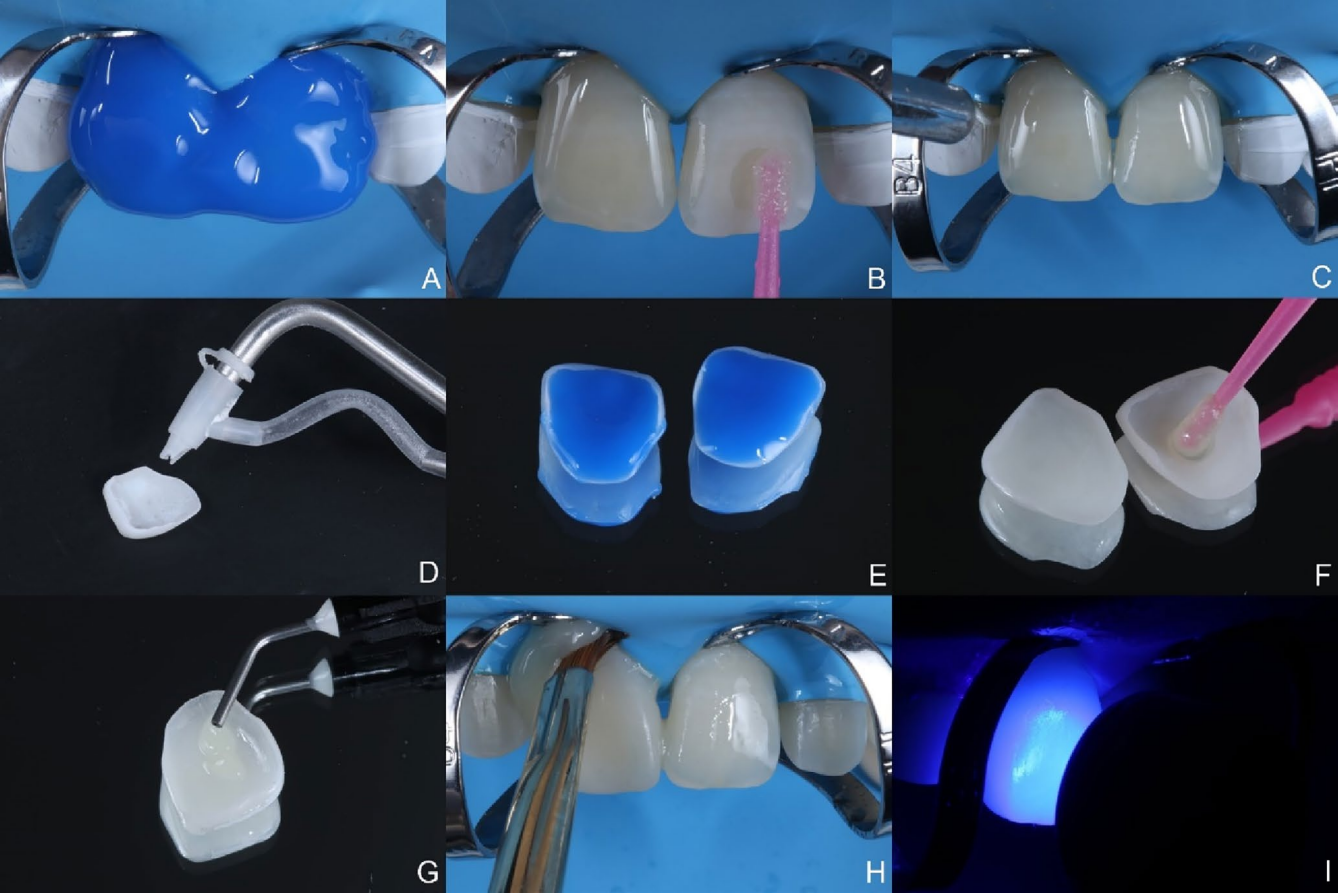
Adhesive cementation protocol. (A) Acid etching of teeth performed with phosphoric acid. (B, C) Application of a hydrophobic adhesive. (D) Sandblasting of the veneer using 29 μm aluminum oxide. (E, F). Treatment with phosphoric acid and a silane coupling agent. (G–I). Veneers were bonded with a light‐cure resin cement under absolute isolation, ensuring adhesion and longevity.

For intraoral trial fitting, a clear try‐in paste (Natures Try‐In, BM4, Maringá, Brazil) was used. Upon confirmation of the adaptation and esthetics, cementation was performed under rubber dam isolation using a heavy rubber sheet (Nic Tone, PHS Group, Joinville, Brazil) and retractors (Brinker B4, Coltene, Cuyahoga Falls, United States) to ensure proper gingival retraction for veneer seating. The teeth were etched with 35% phosphoric acid (Ultra‐Etch, Ultradent, Indaiatuba, Brazil) for 30 s, rinsed, and dried. An adhesive system (Adper Scotchbond Multi‐Purpose, Solventum, Sumaré, Brazil) was applied without light curing. Sandblasting was performed on the veneers using 29 μm aluminum oxide powder (Aquacare, Velopex, London, England), followed by rinsing and etching with 35% phosphoric acid (Ultra‐Etch, Ultradent, Indaiatuba, Brazil) for 60 s to remove residues. A silane coupling agent (Monobond N, Ivoclar Vivadent, Schaan, Liechtenstein) was applied for 60 s and dried. A light‐cure resin cement in clear shade (Natures Veneer, BM4, Maringá, Brazil) was applied to the veneers, carefully seated, and excess cement was removed with a brush and dental floss. Light curing was performed (Valo Grand, Ultradent, Indaiatuba, Brazil) for 40 s (Figure 8). Occlusal adjustments were made in maximum intercuspation and excursive mandibular movements using articulating paper (Progress 100, Baush, Nashua, United States) and diamond polishing rubbers (Eve H8, Odontomega, Ribeirão Preto, Brazil). The composition of materials used for the fabrication and bonding procedures of veneers, as described by the manufacturers, can be seen in Table 1.

**TABLE 1:** Materials used for the fabrication and bonding procedures of veneers as described by the manufacturers.

Material	Composition (% by weight)	Manufacturer
Prizma 3D Bio Crown	Methacrylate Monomers (> 10%), Amorphous Silica (≤ 8%), Urethane Dimethacrylate (UDMA, < 75%), Titanium Dioxide (< 0.5%), Silanized Zirconia (< 2%), Ceramic Fillers (< 15%), Diphenyl (2,4,6‐Trimethylbenzoyl)‐Phosphine Oxide (TPO, < 5%)	Makertech Labs, Tatuí, Brazil
Prizma SEAL	Monomers (< 50%), Photoinitiators (< 10%)	Makertech Labs, Tatuí, Brazil
Ultra‐Etch	Phosphoric Acid Solution (< 40%), Polyethylene Glycol (> 2.5% ≤ 10%), Dimethicone (< 1%)	Ultradent, Indaiatuba, Brazil
Adper Scotchbond Multi‐Purpose	Bisphenol A Diglycidyl Ether Dimethacrylate (Bis‐GMA, 55%–65%), 2‐Hydroxyethyl Methacrylate (HEMA, 35%–45%)	Solventum, Sumaré, Brazil
Monobond N	Alcohol Solution Of Silane Methacrylate, Phosphoric Acid Methacrylate, Sulphide Methacrylate	Ivoclar Vivadent, Schaan, Liechtenstein
Natures Veneer	Methacrylate Monomers, 2‐Hydroxyethyl Methacrylate (HEMA), Photoinitiators, Preservative, Stabilizer, Fillers, Pigments	BM4, Maringá, Brazil

### 2.6 Final Evaluation

**FIGURE 9:**
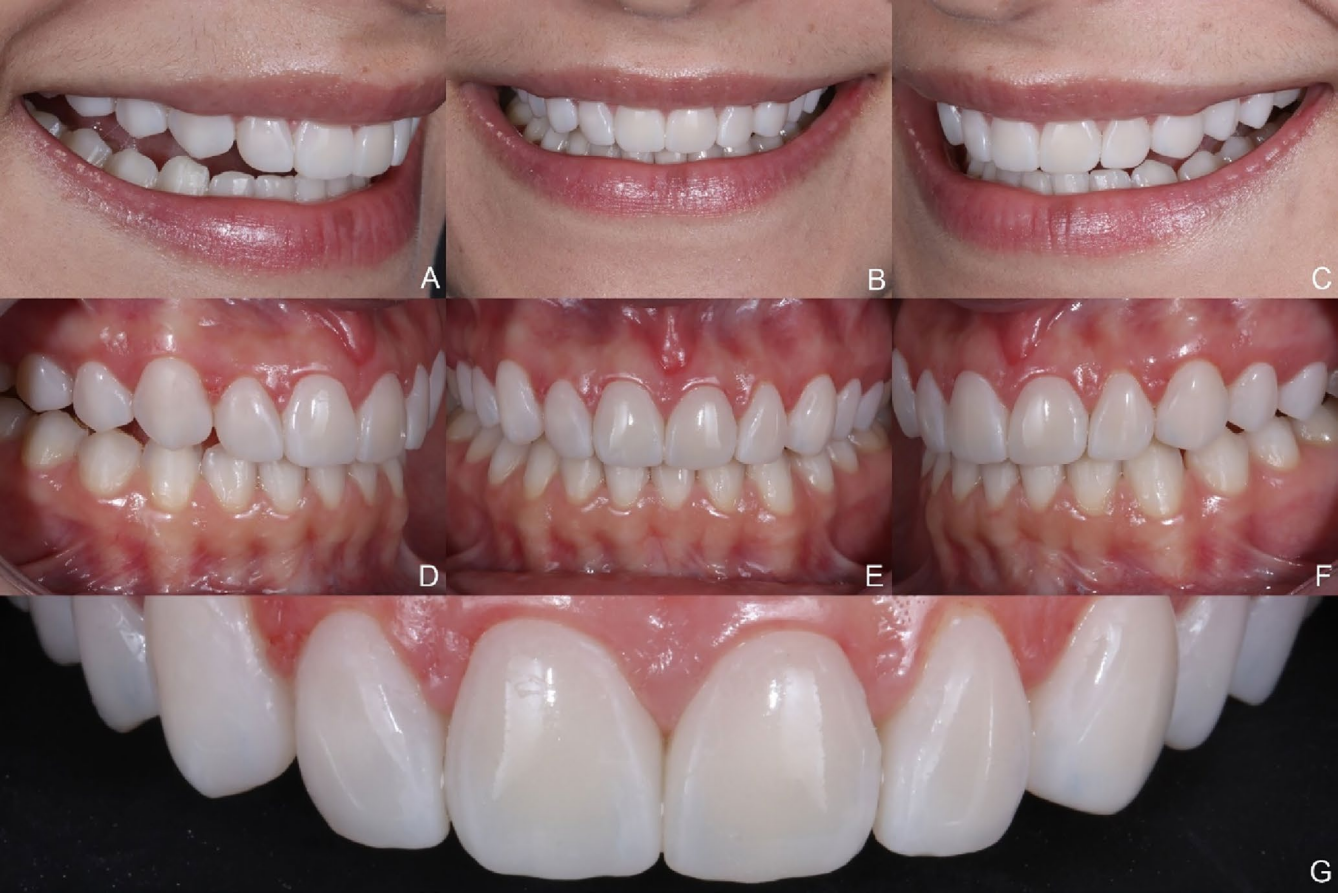
(A to G) Final extraoral and intraoral photographs. Esthetics were improved and met the patient's expectations.

**FIGURE 10:**
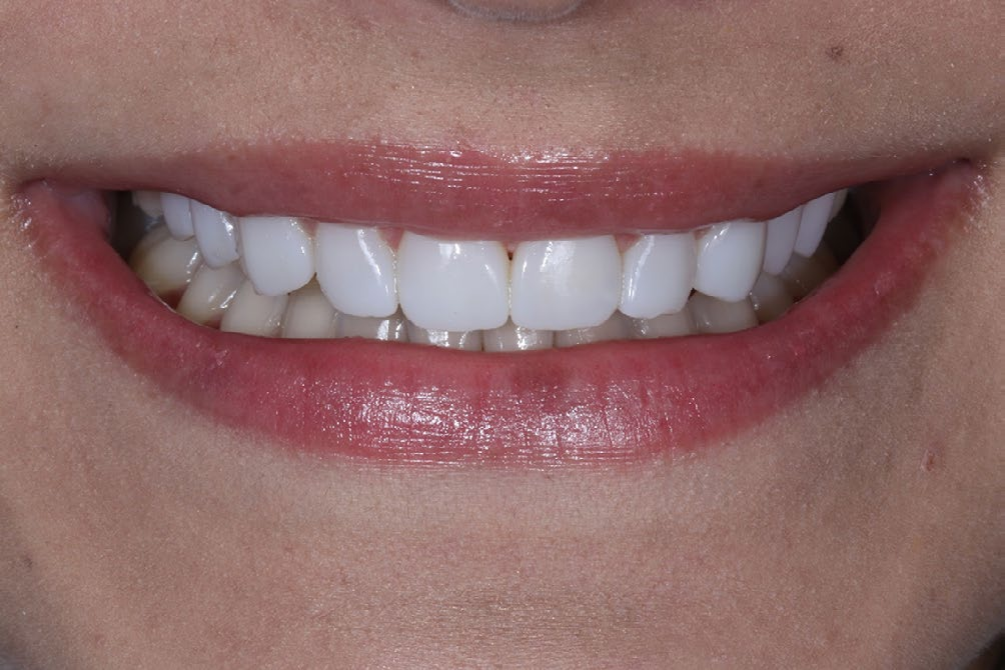
Extraoral view at the two‐year follow‐up.

**FIGURE 11:**
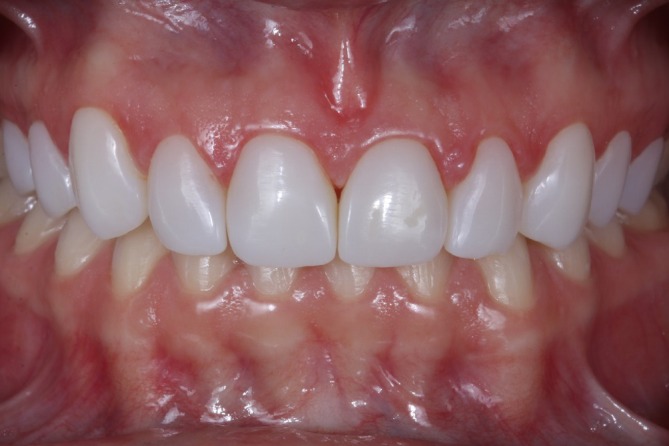
Intraoral view at the two year follow‐up.

**FIGURE 12:**
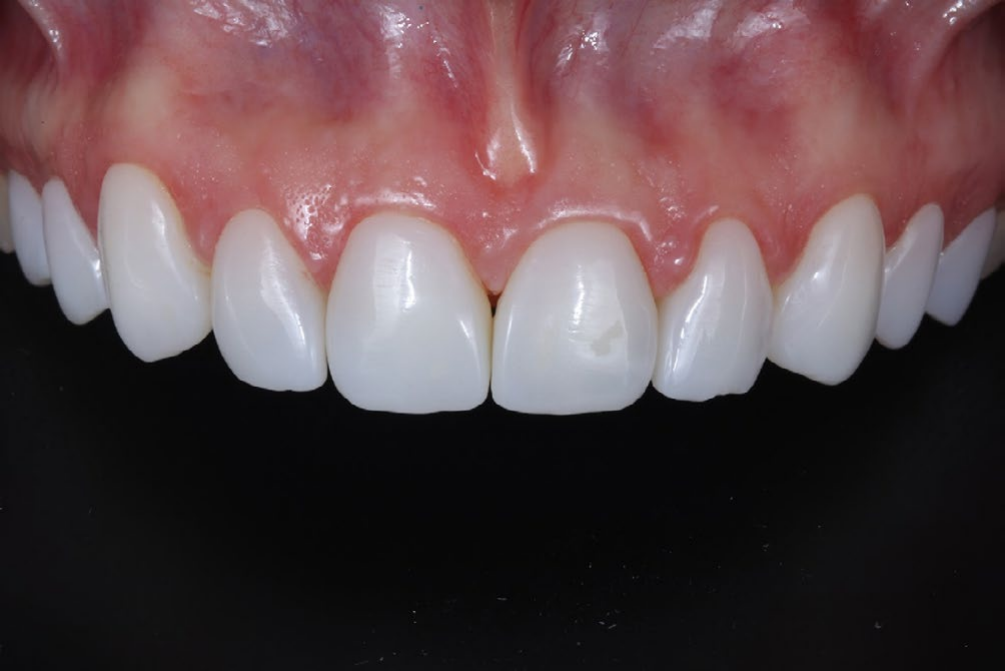
Intraoral view at the two‐year follow‐up. Incisal chipping of the maxillary left lateral incisor is observed. Color and esthetic outcomes remain stable.

The immediate post‐cementation results demonstrated esthetic parameters that met the patient's expectations (Figure 9). A follow‐up after two years confirmed case stability and color stability. However, chipping was observed at the incisal edge of the maxillary left lateral incisor (Figures 10, 11, 12).

## 3 Discussion

Dental veneers are a minimally invasive treatment option for various conditions, including tetracycline‐stained teeth, fluorosis, amelogenesis imperfecta, aging, fractured, worn, abnormally shaped teeth, or minor misalignments [5, 6]. Traditionally, ceramic veneers are employed for these clinical situations. However, the digital revolution has significantly transformed dental practice, and the use of 3D printing for the fabrication of resin veneers has shown advantages, such as production speed, reduced material waste, and lower costs compared with subtractive methods [7, 10, 14, 15].

In the present study, veneers made from biocompatible nanohybrid resin suitable for 3D printing, utilizing an additive manufacturing technique, were fabricated to improve the esthetics of a 24‐year‐old female patient's teeth. Initially, the patient underwent a photographic protocol and arch scanning to obtain the restorative planning. Intraoral scanners are widely used digital devices for model acquisition and treatment planning. When combined with a photographic protocol, these devices help optimize planning time, providing more efficient diagnoses, consistent treatment plans, and improved outcomes [16].

One of the advantages of a digital workflow is the ability to generate a preoperative mock‐up [16]. The esthetic mock‐up is an intraoral representation of the treatment plan, simulating the final contour of the teeth post‐treatment [4]. This procedure allows the patient to visualize the impact of the new smile before committing to the treatment and irreversible procedures, thereby enhancing patient understanding and case acceptance [4, 16]. In this case, two options of esthetic mock‐ups with distinct designs were fabricated using additive manufacturing and tested intraorally to allow the patient to choose the preferred design. After evaluating both designs, the patient expressed a preference for the one featuring more subtle and rounded incisal angles. The ease of insertion and removal of each mock‐up during the trial phase enabled a comprehensive assessment by both the patient and clinician, facilitating the precise determination of the final restorations. However, in treatment plans involving prior dental reduction, the mock‐up will not provide adequate intraoral adaptation.

Subsequently, the teeth underwent minimally invasive preparations on the buccal and incisal surfaces to regularize edges and achieve an appropriate emergence profile. The primary goal of tooth preparation is to create sufficient space for the restorative material to exhibit excellent esthetics and fracture resistance during function. Literature suggests that minimal tooth structure reduction promotes better adhesion and clinical longevity, as mechanical interlocking with enamel provides more stable adhesion than bonding to dentin [4].

Regarding the fabrication of final restorations, CAD/CAM technologies are widely employed. Currently, these restorations can be created using both subtractive and additive techniques, with the latter gaining popularity. A previous study observed that crowns fabricated by 3D printing demonstrated higher precision with fewer marginal gaps compared with milled crowns [17]. Additionally, 3D printing was highlighted for its advantage in producing complex shapes with undercuts or areas that cannot be fabricated or are limited by the milling process [18]. The additive technique uses specific composite resins developed for 3D printing, which must possess suitable rheological and optical properties. These characteristics are often achieved by reducing the inorganic filler content in the resins, as increased filler concentration can elevate viscosity, reducing flowability. Additionally, filler particles may absorb light, decreasing the thickness that can be polymerized in each layer [13]. According to the manufacturer, the resin used in this case was developed to provide adequate esthetic and mechanical properties for both anterior and posterior teeth. However, the literature indicates that 3D‐printed resins exhibit inferior mechanical properties compared with milled resins [8].

Dental appliances are subjected to various stresses in the oral cavity, such as chewing forces and parafunctional habits [19]. In this case, chipping at the incisal edge of the maxillary left lateral incisor was observed. The patient reported that the fracture occurred ~1 year after the veneers were placed, during the act of biting a nut. This failure may be associated with the aforementioned inferior mechanical properties of 3D‐printed composite resins compared with milled resins, which may have compromised the resistance of the material to functional loads. A previous study showed that the printing nature and photopolymerization of 3D‐printed resin are the main causes for lowering its strength in comparison to milled and conventional resins [20].

Factors influencing the flexural strength of these materials can be categorized into three stages: pre‐printing, which includes the incorporation of reinforcing agents; printing, encompassing parameters such as printing orientation, layer thickness, third‐party resin usage, and object positioning on the build platform; and post‐printing, which involves post‐curing conditions (time, temperature, and curing unit), rinsing duration, finishing and polishing techniques, and storage conditions [19]. Another study assessed the marginal adaptation and fracture resistance of milled and 3D‐printed CAD/CAM hybrid dental crown materials with varying occlusal thicknesses. The findings indicated that CAD/CAM hybrid material crowns exhibit favorable marginal adaptation and fracture resistance, even at a minimal occlusal thickness of 0.8 mm. However, occlusal thickness was shown to significantly influence fracture resistance [21]. In the present clinical case, the veneers had a thickness of ~0.4–0.7 mm, which may have influenced fracture resistance and contributed to the chipping in the lateral incisor.

For definitive restorations, it is crucial that the material possesses satisfactory mechanical properties, wear resistance, a high degree of conversion, and biocompatibility. Additionally, the surface should be smooth and homogeneous to prevent plaque accumulation and staining while minimizing wear on both the restoration and the opposing dentition. Excessive wear can compromise the longevity and functionality of the restoration, potentially leading to material degradation, loss of anatomical shape, and increased risk of fractures. In this context, wear resistance is one of the most important factors in ensuring the functionality of long‐term restorations in the oral cavity [22].

A previous study reported that the amount of filler component in 3D‐printed materials can significantly influence their wear behavior [23]. However, when a substantial amount of filler is introduced into the 3D printing material mix, the viscosity increases, impairing the capability of the material to flow uniformly between the platform and the vat, leading to incomplete object formation, as previously mentioned [22]. Another study compared the wear of 3D printing materials with milling and conventional resin opposed by zirconia and metal. Materials for 3D printing did not show a significant difference in the maximal wear depth or the volumetric loss compared with milled and conventional resin materials [24]. Additional research evaluated the wear resistance of different 3D printing materials against stainless‐steel antagonists and found that all materials demonstrated significant wear resistance. The coefficient of friction (CoF) remained consistently low (below 0.3) across all tested groups, indicating favorable tribological properties for dental applications. Despite this, the study observed an increase in surface roughness after wear testing. However, this increase in roughness did not correspond to an increase in the CoF, suggesting that wear resistance was maintained despite surface changes. These findings highlight that additive manufacturing materials, while showing effective wear resistance, may still undergo surface alterations that could affect other properties, such as bacterial adhesion. Furthermore, these changes in surface roughness can significantly influence the esthetic appearance of 3D‐printed materials [25]. A previous study tested the surface roughness, microhardness, and color change values of 3D‐printed permanent resins and resin‐based CAD/CAM blocks, and the findings showed that the 3D‐printed permanent resins exhibited more color changes than the resin‐based CAD/CAM blocks. Moreover, the color change increased with the storage time in tea and coffee [26].

The literature reports that discoloration rates depend on material composition, post‐processing, and surface treatment [27]. A prospective study evaluated the color stability of 3D‐printed restorations over 24 months in vivo, compared with conventional restorations. Color measurements were taken at 6, 12, and 24 months using a spectrophotometer, and the authors concluded that 3D‐printed restorations exhibit reduced color stability over time, compared with conventional restorations, and are recommended for use within 6 months [28]. In this case, after a two‐year follow‐up period, the 3D‐printed veneers demonstrated remarkable clinical stability, with no observable wear or visible esthetic changes. The optical characteristics of the restorations, including color and gloss, were consistently maintained, and the patient remained satisfied with the results throughout this period. Furthermore, during the fabrication process, the veneers were adjusted, post‐processed, and finalized with a UV‐curable glaze to reduce porosities, seal the resin surface, enhance abrasion resistance, and achieve the desired gloss. These factors may have contributed to the maintenance of the optical characteristics of the restorations.

The outcomes of this study met the patient's esthetic demands, with clinical stability observed after 2 years. However, as this technology is newly introduced into dentistry, clinical data on partial coverage restorations lack sufficient evidence, limiting the validation of their clinical relevance. Moreover, several critical factors must be considered, including material composition and the specifics of printing systems and associated technologies. Longer follow‐up periods and clinical studies are required to confirm these findings in the long term.

## 4 Conclusions

Compared with subtractive manufacturing, additive manufacturing enables the simultaneous production of complex restorations with greater efficiency, reduced material waste, and lower costs. This approach may be a viable treatment alternative, as demonstrated in this clinical case, which showed satisfactory short‐term results with a 2‐year follow‐up. However, longer follow‐up periods and clinical studies are required to confirm these findings in the long term.

## Disclosure

The authors have nothing to report.

## Conflicts of Interest

The authors declare no conflicts of interest.

## Data Availability

Data sharing not applicable to this article as no datasets were generated or analysed during the current study.
